# Routine Detachment of the Anterior and Septal Tricuspid Leaflets Simplifies VSD Closure and Improves the Outcomes

**DOI:** 10.3390/medicina58121849

**Published:** 2022-12-15

**Authors:** Rodrigo Sandoval Boburg, Christian Schlensak, Michael Hofbeck, Harry Magunia, Rafal Berger, Walter Jost, Migdat Mustafi

**Affiliations:** 1 Department of Thoracic and Cardiovascular Surgery, University Hospital Tuebingen, Eberhard-Karls-University Tuebingen, Hoppe-Seyler-Str. 3, 72076 Tuebingen, Germany; 2 Department of Pediatric Cardiology and Intensive Care Medicine, University Hospital Tuebingen, Eberhard-Karls-University Tuebingen, Hoppe-Seyler-Str. 3, 72076 Tuebingen, Germany; 3 Department of Anaesthesiology and Intensive Care Medicine, University Hospital Tuebingen, Eberhard-Karls-University Tuebingen, Hoppe-Seyler-Str. 3, 72076 Tuebingen, Germany

**Keywords:** congenital surgery, ventricular septal defect, tricuspid valve detachment

## Abstract

*Background and Objectives:* The closure of perimembranous ventricular septal defects (VSDs) is one of the most common surgeries performed in infancy. The technique of detachment of the anterior and septal leaflets of the tricuspid valve (TV) with subsequent leaflet augmentation is frequently used for isolated as well as non-isolated VSD closure. In this study, we compared the incidence of tricuspid regurgitation (TR) in patients who underwent a VSD repair with and without detachment of the TV in the short- and long-term follow-up. *Materials and Methods:* A retrospective study that included 140 patients who underwent perimembranous VSD closure at our center from 2011–2016, where 102 of these patients underwent the procedure with detachment of the TV, was performed. The follow-up data were obtained from postoperative echocardiography performed in the follow-up visits. A total of 62 patients underwent follow-up at our center, where the follow-up time ranged from 1 to 9 years, with a mean of 71 ± 2.47 months. *Results:* Regarding patients who underwent a VSD repair with a detachment of the TV, 98.1% of the patients had none to mild TR, compared to 94.7% in patients without intraoperative TV detachment at the time of discharge. There were no reported cases of moderate to severe TR, atrioventricular blocks, aortic insufficiency, or deaths. A total of 98.1% of patients who underwent follow-up at our center with a TV detachment had none to mild TR compared to 94.7% in the group without TV detachment. *Conclusion:* TV detachment with leaflet augmentation for VSD closure is safe and effective and does not increase the incidence of TR in the short- and long-term follow-up.

## 1. Introduction

Ventricular septal defect (VSD) is the most common diagnosed congenital heart defect, with an incidence of 40–48% [1,2]; this, however, entails all types of VSDs, many of which do not require any treatment due to spontaneous closure in the first years of life, as well as larger ones that require surgical or interventional closure [1,3,4]. Perimembranous VSDs are the most frequent defects among those requiring surgical closure [5]. Since the closure of the first VSD in the University of Minnesota in the 1950s, reports have emerged throughout the years with satisfactory postoperative results; despite the good results, after all of these years, there are groups who report a high incidence of rest VSD after closure [6,7,8]. We believe one of the main factors that influence the result is the exposure of the VSD. Cardiac structures such as the aortic valve, atrio-ventricular (AV) node, and the tricuspid valve are close to the borders of VSDs, and, if not examined carefully, may be affected by the patch-closure of the defect [9,10].

Throughout the years, different surgical techniques have been refined, among other things, to achieve a better exposure of the VSD. Hudspeth and colleagues were the first to describe a transatrial approach for the repair of a perimembranous VSD, in which the septal leaflet of the tricuspid valve (TV) is detached at the annulus [11]. After detachment, the borders of the VSD were recognized, and a closure with a bovine pericardium or dacron patch was possible; after closure, the TV was re-attached [11].

Although a TV detachment (TVD) is not routinely used in many centers, several groups have used this technique and proved its efficacy in different groups of patients. Recent literature has shown that detachment of the septal leaflet is a safe procedure in children under 3 months, those who weigh less than 5 kg, those who have abnormal chord attachment as seen in echocardiography, and those who have a VSD that is difficult to visualize [9,10,12,13,14,15]. These studies have shown good results in long-term follow-up of up to 7 years [9,10,12]. The TVD technique described by most groups mainly focuses on the detachment of the septal leaflet and, rarely, the anterior leaflet; after the closure of the VSD, the TV is directly sutured to the tricuspid annulus [9,10,14,15,16]. We took this technique and modified it to achieve a better exposure of the VSD and diminish the risk of developing a TV regurgitation. We routinely detached the anterior tricuspid leaflet and extended the incision to the septal leaflet if necessary, and, instead of directly re-attaching the valve to the annulus, we performed a leaflet augmentation using an autologous pericardium.

The objective of this study was to compare the incidence of tricuspid regurgitation (TR) (short- and long-term), atrioventricular (AV) block, residual VSDs, and reoperations following the transatrial closure of a perimembranous VSD as a main diagnosis or part of a complex anomaly among patients who underwent TVD with leaflet augmentation and those who did not.

## 2. Methods

### 2.1. Patient Selection Process

**Figure 1:**
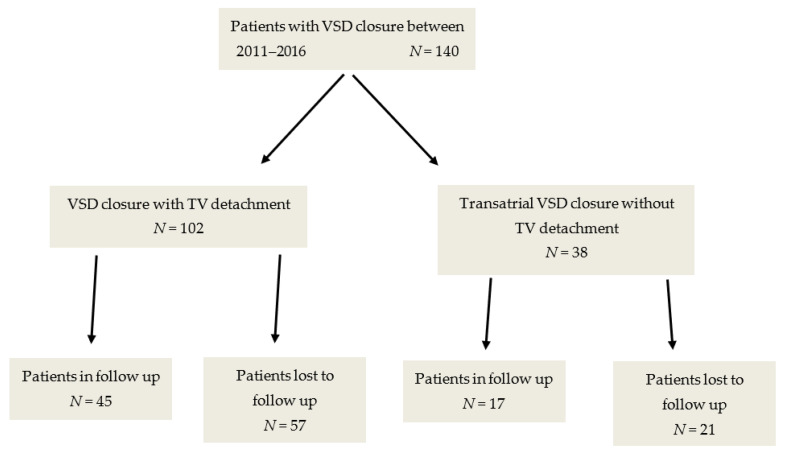
Patient and group selection. TV: tricuspid valve; VSD: ventricular septal defect.

This retrospective study included all patients who underwent transatrial repair of perimembranous VSD, either as main surgery or part of a complex surgery, in our center between 2011–2016. The surgeries were performed by the same group of surgeons. A total of 140 patients were included in this study: 102 patients underwent surgery with TVD and 38 patients underwent conventional transatrial closure. The decision to perform TVD was made intraoperative by the surgical team depending on whether all borders of the VSD were clearly visible and suitable for closure. A total of 62 patients were followed up at our center. Long-term follow-up was defined as availability of postoperative echocardiographic examinations beyond 24 months after surgery. Patient selection is shown in Figure 1.

### 2.2. Operative Technique

After performing a median sternotomy, standard bi-caval cannulation for cardiopulmonary bypass (CPB), and cold cardioplegic arrest, an incision was made in the right atrium and a vent was placed in the left atrium through the fossa ovalis.

The VSD was first inspected through the TV. If the entire rim was identified and closure was possible without compromising the aortic or tricuspid valve, closure was performed without detaching the valve. However, if the borders of the VSD, especially close to the aortic valve, could not be properly identified, the decision was made to detach the TV.

**Figure 2:**
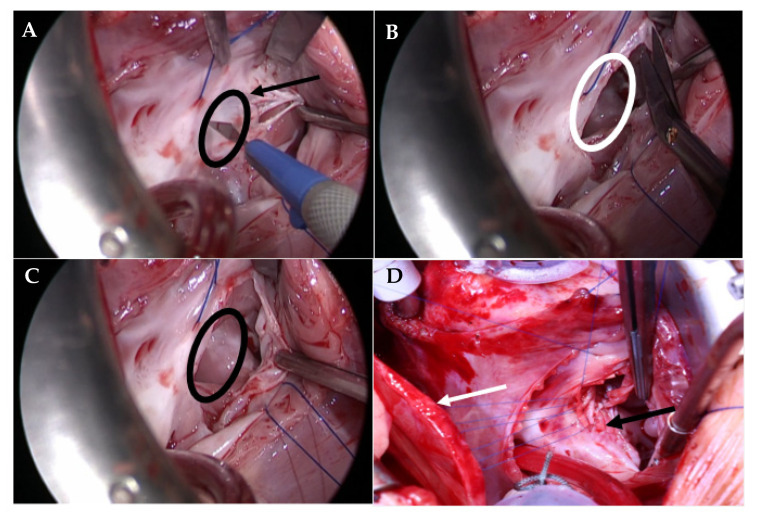
(**A**) Detachment of the anterior leaflet of the TV right above the VSD; the arrow is pointing to the anterior TV leaflet and the circle shows the location of the VSD. (**B**) The incision is extended to the septal leaflet to obtain a better exposure of the VSD. (**C**) Complete exposure of the VSD is achieved after detaching and retracting the anterior and septal TV leaflets. (**D**) The VSD is closed using bovine pericardium (black arrow). In the region close to the AV node, the suture line is strengthened with autologous pericardium (white arrow). The pericardium will later be used to perform a leaflet augmentation. TV: tricuspid valve, VSD: ventricular septal defect.

An incision was made in the anterior leaflet of the TV at the annulus, where the extent of the detachment of the septal leaflet depended on the size and location of the VSD. If necessary, the detachment was continued all the way through the septal leaflet to enable optimal visualization of the defect. The valve was carefully pulled aside with a nerve hook for the duration of the repair (Figure 2). VSD closure was performed using bovine pericardial or dacron patch and a running polypropylene 6-0 suture (Figure 2).

**Figure 3:**
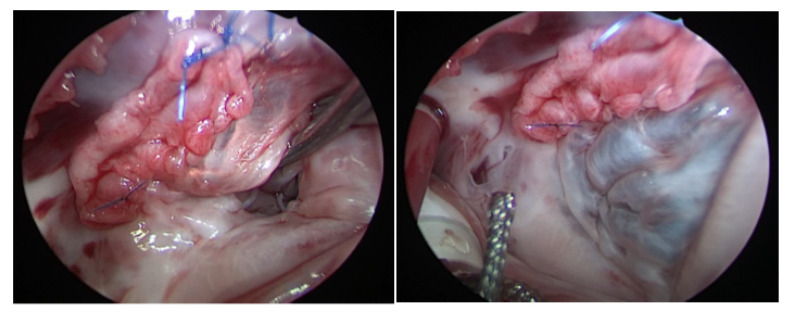
(**Right**) TV after reconstruction and augmentation of the leaflets, the black arrow is pointing to the pericardial patch used to perform the augmentation. (**Left**) TV after injecting water in the RV for the water probe showing a competent TV. RV: right ventricle, TV: tricuspid valve.

When placing the sutures along the TV annulus where the leaflets were detached, the sutures were placed superficially, in order to avoid injuring the AV-node, and strengthened with autologous pericardium. After closure of VSD was completed, we performed an augmentation of the septal and/or anterior leaflet with autologous pericardium and reattached it to the annulus using a polypropylene 6-0/7-0 running suture (Figure 3). After completing the reattachment, the TV was tested with cold saline injection into the right ventricle. If a significant regurgitation was present, a TV reconstruction was performed; if the TV was competent, atrium closure using 6-0 polypropylene suture in Blalock technique was performed, and disconnection from CPB and sternum closure were carried out as usual (Figure 3).

### 2.3. Echocardiographic Studies

Transthoracic echocardiography (TTE) was performed in all patients prior to surgery. Intraoperatively, after CPB was terminated, a transesophageal echocardiography (TEE) was performed by a pediatric cardiologist to check the surgical result, with a special focus on complete closure of the VSD and competency of the TV. Postoperative TTE studies were performed on a regular basis at the pediatric intensive care unit prior to referral of the patients to the normal ward and prior to discharge from the hospital. During follow-up, the TV was evaluated using echocardiographic parameters defined by the American Society of Echocardiography and was classified into mild, moderate, and severe [17]. The preoperative echocardiographic studies were compared to the intra- and postoperative examinations to determine if there was a significant change in TR.

### 2.4. Statistical Analysis

All statistical analyses were performed using SPSS 23.0 (IBM Corporation, Armonk, NY, USA) software. Patient characteristics and surgical data were tested for a normal distribution with a Kolmogorov–Smirnov test; normally distributed data were compared using a student *t*-test. Data that did not follow a normal distribution were analyzed using a Mann–Whitney U-test. The patients were separated into two groups according to the surgical technique: with and without TV detachment. Pre- and postoperative results were compared between both groups using Fisher’s exact test.

### 2.5. Written Consent

The ethics committee of the University of Tübingen approved this study 280/2020BO1. Because of its retrospective nature, written consent was waived.

## 3. Results

We included a total of 140 patients: 102 (72.8%) underwent VSD repair with TVD, whereas 38 (27.2%) underwent conventional transatrial closure. The demographic characteristics among both groups were similar: there was no significant difference when comparing the age or weight of the patients between groups. The youngest patient was 11 days old at the time of surgery and the urgency was due to an accompanying aortic isthmus stenosis; the oldest patient was 9.6 years, and this patient moved to Germany with his family from a foreign country. Therefore, a late diagnosis was performed. Isolated perimembranous VSD was the most common diagnosis in our cohort, followed by tetralogy of Fallot. Five patients required a reconstruction of the aortic arch due to aortic coarctation and hypoplastic or interrupted aortic arch, and an additional two patients required an arterial switch operation. A comparison of the cross-clamp and cardiopulmonary bypass (CPB) time between both groups revealed no significant difference in either of these parameters (Table 1).

Of the patients who underwent the VSD repair with TVD, 8 (8.8%) had mild TR preoperatively; the postoperative TEE control showed a mild TR in 31 (30.4%) of the patients, 1 (1%) showed a moderate TR, and there were no patients with severe TR. At the time of discharge, patients underwent another TTE. This showed a mild TR in 34 (33.3%) of patients and moderate TR in 2 (%) of them.

In the group without TVD, 3 (7.9%) patients showed a mild TR and 1 (2.6%) showed a moderate preoperative TR; 3 (7.9%) and 2 (5.3%) patients showed a mild and moderate TR intraoperatively; at the time of discharge, 14 (36.8%) of the patients showed a mild TR and 2 (5.3%) showed a moderate TR. None of the patients in this group showed severe TR.

We performed a subgroup analysis in each group (TVD and no TVD) of patients with follow-up at our center: we sub-divided the patients into those who underwent VSD closure as a main surgery and those who had it as a part of a more complex surgery. In the group without TVD, there were 11 patients who had VSD closure as a concomitant surgery. A total of 3 (27.2%) patients had a postoperative TI compared to 5/8 (62.5%) patients who only had a VSD closure (*p*-value 0.18). In the patients with TVD, 23 underwent a concomitant VSD closure and 10 (47.6%) had a mild postoperative TI; 24 patients underwent VSD closure alone and 3 (12.5%) had a postoperative TI (*p*-value 0.01).

A total of 19 (18.6%) TV reconstructions were performed after the re-attachment of the TV in the TVD group: 2 leaflet-plasties due to redundant leaflet tissue, 2 annuloplasties, 1 papillary muscle re-implantation, and 14 antero-septal leaflet cleft closures (Table 2).

None of the patients in the entire cohort showed a severe TR pre- or postoperatively. There was one residual VSD in the non-TVD group, one patient in each group showed a postoperative third-degree AV-block requiring a pacemaker, and one patient in the non-TVD group died in the postoperative phase.

We performed a subgroup analysis in patients under 3 months of age. There was a total of 26 patients: 15 in the TVD group and 11 in the non-TVD group. A total of 6 (40%) patients in the TV detachment group showed mild TR at the time of discharge compared to 6 (54.5%) in the non-TV detachment group (*p*-value = 0.46).

A total of 48 (34.3%) patients weighed under 5 kg at the time of surgery in this cohort: 33 in the TVD and 15 in the non-TVD group. None of the patients showed moderate or severe TR in the TVD group and only one patient showed a moderate TR in the non-TVD group. Four patients (12.1%) in the TVD group required antero-septal leaflet cleft closures, and one patient (3%) required papillary muscle re-implantation.

Following discharge, 62 (44.3%) of the patients were examined at our institution for at least two years, with a maximum follow-up period of 9.6 years. The mean follow-up period among these patients was 71 ± 2.5 months. A total of 45 (72.6%) patients underwent closure with TVD. A total of 32 (71.1%) of these patients showed no TR, 13 (28.9%) showed a mild TR, and none of the patients had a moderate TR. Among the patients who underwent surgery without TVD, 10 (58.8%) showed no TR, 6 (35.3%) patients were diagnosed with mild TR, and 1 (5.9%) with moderate TR (Table 3). A total of 15 patients under 3 months of age underwent follow-up at our center: 10 from the TV detachment group and 5 from the non-TV detachment group. Only 3 (30%) patients in the TV detachment and 1 (20%) in the non-TV detachment group showed mild TR, and all others had no TR during follow-up.

There were no re-interventions recorded in this patient cohort (Table 3).

## 4. Discussion

When comparing the demographic data in this patient cohort, there was no difference regarding gender, age, and weight at the time of surgery between both groups. We also did not identify any differences regarding the CPB or aortic cross-clamp time between both groups in our study. This finding differs from previous studies that showed that patients who underwent VSD closure with TVD had significantly longer CPB and aortic cross-clamp times when compared to patients without TVD [18]. This difference may be due to the surgeon’s experience with this technique; during the study period, the surgeries were mainly performed by a single experienced surgeon.

In recent years, various study groups seem to have settled the debate about whether it is safe to detach the TV for perimembranous VSD repair. Fraser et al. and Lucchese et al. performed retrospective studies with long-term follow up (over 5 years) with good results, proving that this is a safe procedure for isolated VSD closure [9,10]. Bilen et al. and Bang et al. performed a similar study but focused on patients who weighed less than 3 kg; their findings showed that TVD, with the right indication, is safe in this patient population [13,14]. Lee et al. showed in a recent publication that, in patients with isolated VSDs weighing under 5 kg, TVD is a safe procedure as well [12]. Although there is close to no incidence of severe TR, AV-blocks, or aortic regurgitation, the rate of residual VSDs remains high even after TVD [8,9,10,12,13,14,15,16]. We believe that this rate of residual VSDs may be due to a suboptimal exposure of the defect, especially underneath the anterior TV leaflet.

The current debate focuses on the indications for TVD rather than on its safety. Until now, TVD has been described as a detachment of the septal leaflet and has been mainly used for isolated VSD closures; the described indications have been an abnormal chord attachment and difficulty in visualizing and/or reaching all borders of the VSD [13,14,16,18].

Our cohort mainly included patients with isolated VSDs, but also patients with tetralogy of Fallot and concomitant congenital defects. Due to the morphology of many VSDs, an adequate visualization may be challenging, which is why we opted for a detachment of the anterior TV leaflet and, if needed, an extension of the incision to the septal leaflet. For the valve re-attachment, we used a technique that has been described for the repair of incompetent TV: a leaflet augmentation using autologous pericardium [19,20]. We believe that, through the leaflet augmentation, we are able to mainly achieve two objectives: the first, avoiding the shortening of the leaflet at the time of re-attachment and creating a greater coaptation surface for a better closure of the valve, thus requiring fewer valvuloplasties; second, due to the strengthened sutures close to the av-node, av blocks can be avoided by placing the sutures rather superficially at the annulus and still having enough support from the pericardium to fixate the leaflet. To date, there have mainly been reports of tricuspid valve augmentation for the treatment of Ebstein’s anomaly, but not after VSD closure [21].

When analyzing the prevalence of preoperative TR, there was no difference between groups, as expected, since the patients in this cohort had no TV anomalies; there was only one patient in the non-TVD group who had a moderate TR preoperatively. The intraoperative echocardiography showed that, after TVD, 99% of patients had none to mild TR, compared to 94.7% in the non-TVD group, without having a longer CPB or cross-clamp time; this fact gains relevance if, additionally, there was no residual VSD, av-block, or deaths in this cohort. This trend remained unchanged at the time of discharge, where 98.1% of patients in the TVD group and 94.7% in the non-TVD group had none to mild TR. These results show that, in the short-term, this technique is effective and safe.

In the first subgroup analysis that we performed, comparing VSD closure alone and as a concomitant surgery, we found that, in the TVD group, the rate of mild TI was significantly higher in patients with a VSD as a concomitant surgery. In patients without TVD, there was no difference between the groups. We could not find any reports in the literature that have addressed this topic. We believe that the increased rate of mild TR in patients after concomitant VSD closure and TVD may be due to manipulation; for example, for muscle bundle resection in patients with tetralogy of Fallot.

We performed two subgroup analyses that included patients who were 3 months old or younger and patients who weighed less than 5 kg; these populations were described as high risk for such procedures and some groups proved that a radial incision and re-attachment of the TV, if performed by experienced surgeons, were possible and safe [12,13,14]. In our analyses, none of the 15 patients that were 3 months old or younger in the TVD group had a moderate or severe TR in either short- or long-term follow-up; of the 33 patients who weighed under 5kg in the TVD group, none had a moderate or severe TR either.

The rate of postoperative complications was comparable with that in the literature. The only patient in the TVD group who suffered from a third-degree av-block was not defined as “patient at risk” due to their age or size [9,10,12]. In the non-TVD group, there was one patient who suffered from third-degree av block, one patient who had a residual VSD, and one patient who died postoperatively, and none of these patients were defined as “at risk” due to their weight or age either.

Unfortunately, we only had follow-up data on 44.3% of the patients in the entire cohort, which is lower than what has been reported by other groups [9,16]. However, we had data on these patients for at least 2 years and a maximum of 9.6 years after surgery. Our data correlate with that of other groups, who showed that there was little to no incidence of moderate or severe TR after TVD after hospital discharge; in our case, there were no patients who suffered from a moderate or higher degree of TR after valve detachment, even after 9 years [9,10,12,13,14,15,16]. This shows that, even at stages where the patients show a rapid growth, re-attachment using leaflet augmentation shows satisfactory results.

## 5. Limitations

This study was subject to several limitations, the first one being its retrospective nature. It was a single-center study with a limited patient population, where the surgical team had a predefined method of choice, making the groups uneven, which may lead to a type II error. Echocardiographic data were obtained from both images and reports; however, images were not available for all patients, and, in these cases, we sometimes had to rely on the reports, especially patients operated on in 2011. Given the fact that different people performed echocardiographies at different times, we cannot account for inter-observer variability. For future studies, we would recommend a larger sample size, with similar groups and standardized postoperative time points at which patients undergo an echocardiography.

## 6. Conclusions

Our results show that TVD of the anterior leaflet with subsequent TV augmentation for VSD closure is an effective and safe procedure regardless of age and weight in pediatric patients. This technique may be used not only for isolated VSDs but also for any congenital anomaly in which a VSD must be repaired.

## Figures and Tables

**Table 1:** Patient description and diagnosis list.

	TVD	No TVD	*p*-Value
No. of patients	102	38	
Age (years)	0.41 (0.31–0.85)	0.38 (0.24–0.82)	0.72
Gender (m)	51 (50.0%)	21 (56.7%)	0.5
Weight (kg)	5.95 (4.7–7.2)	5.7 (3.85–8.65)	0.45
Cross-clamp time (min)	70.5 (49–89) ± 74.45	68.5 (51–91) ± 66.65	0.92
Cardiopulmonary bypass time (min)	95 (66–123)	97 (82–122)	0.37
**Diagnoses**			
VSD	62	19	
Tetralogy of Fallot	36	15	
VSD, hypoplastic aortic arch	1	1	
TGA, VSD	0	1	
TGA, VSD, ISTA	1	0	
VSD, ISTA	1	0	
PA-VSD	1	2	

ISTA: aortic isthmus stenosis; PA: pulmonary atresia; TGA: transposition of the great arteries; VSD: ventricular septal defect.

**Table 2:** Pre-, intra-, and postoperative tricuspid regurgitation in both groups.

Preoperative TR			
Severity of TR	TVD	No TVD	*p*-Value
**0**	93 (91.2%)	34 (89.5%)	**0.75**
**1**	9 (8.8%)	3 (7.9%)	**1**
**2**	0	1 (2.6%)	**1**
**3**	0	0	**1**
**Intraoperative TR**			
**0**	70 (68.6%)	33 (86.8%)	0.03
**1**	31 (30.4%)	3 (7.9%)	<0.01
**2**	1 (1%)	2 (5.3%)	0.1
**3**	0	0	1
**TR Prior to Discharge**			
**0**	66 (64.7%)	22 (57.9%)	0.46
**1**	34 (33.3%)	14 (36.8%)	0.7
**2**	2 (1.9%)	2 (5.3%)	1
**3**	0	0	1

TVR: tricuspid regurgitation; TVD: tricuspid valve detachment.

**Table 3:** Tricuspid regurgitation in patients during follow-up.

Tricuspid Regurgitation during Follow-Up			
TR Severity	TVD	No-TVD	*p*-Value
**0**	32 (71.1%)	10 (58.8%)	0.36
**1**	13 (28.9%)	6 (35.3%)	0.62
**2**	0	1 (5.9%)	1
**3**	0	0	1

## Data Availability

Due to sensitive patient information data cannot be published, but is available upon request.
